# Hepatitis B virus X protein promotes liver cell pyroptosis under oxidative stress through NLRP3 inflammasome activation

**DOI:** 10.1007/s00011-020-01351-z

**Published:** 2020-04-28

**Authors:** Wen-hui Xie, Jian Ding, Xiao-xia Xie, Xiao-huang Yang, Xiao-Fan Wu, Zhi-xin Chen, Qi-lan Guo, Wen-yu Gao, Xiao-zhong Wang, Dan Li

**Affiliations:** 1 Department of Gastroenterology, Union Hospital of Fujian Medical University, 29, Xinquan Road, Gulou, Fuzhou, 350001 Fujian People’s Republic of China; 2 Department of Gastroenterology, The First Affiliated Hospital of Fujian Medical University, Fuzhou, 350005 Fujian People’s Republic of China; 3 Graduate School, Fujian Medical University, Fuzhou, 350001 Fujian People’s Republic of China

**Keywords:** Hepatitis B virus X protein, Pyroptosis, NLRP3 inflammasome, Liver inflammation

## Abstract

**Objective:**

Hepatitis B virus X protein (HBx) is a pivotal factor for HBV-induced hepatitis. Herein, we sought to investigate HBx-mediated NLR pyrin domain containing 3 (NLRP3) inflammasome activation and pyroptosis under oxidative stress.

**Methods:**

The effect of HBx on the NLRP3 inflammasome was analyzed by enzyme-linked immunosorbent assays, quantitative reverse transcription-polymerase chain reaction, western blotting, and immunofluorescence in hepatic HL7702 cells. Pyroptosis was evaluated by western blotting, lactate dehydrogenase release, propidium iodide staining, and transmission electron microscopy. NLRP3 expression in the inflammasome from liver tissues was assessed by immunohistochemistry.

**Results:**

In hydrogen peroxide (H_2_O_2_)-stimulated HL7702 cells, HBx triggered the release of pro-inflammatory mediators apoptosis-associated speck-like protein containing a CARD (ASC), interleukin (IL)-1β, IL-18, and high-mobility group box 1 (HMGB1); activated NLRP3; and initiated pro-inflammatory cell death (pyroptosis). HBx localized to the mitochondria, where it induced mitochondrial damage and production of mitochondrial reactive oxygen species (mitoROS). Treatment of HL7702 cells with a mitoROS scavenger attenuated HBx-induced NLRP3 activation and pyroptosis. Expression levels of NLRP3, ASC, and IL-1β in liver tissues from patients were positively correlated with HBV DNA concentration.

**Conclusions:**

The NLRP3 inflammasome was activated by elevated mitoROS levels and mediated HBx-induced liver inflammation and hepatocellular pyroptosis under H_2_O_2_-stress conditions.

**Electronic supplementary material:**

The online version of this article (10.1007/s00011-020-01351-z) contains supplementary material, which is available to authorized users.

## Introduction

Hepatitis B virus (HBV) is an oncogenic virus presently responsible for approximately 350 million cases of chronic infections worldwide [1]. HBV’s persistence is reportedly associated with an increased risk of cirrhosis and hepatocellular carcinoma (HCC) [2]. Specifically, the hepatitis viral X protein (HBx), encoded by the *HBV X* gene, is implicated in HBV-related hepatitis, cirrhosis, and the initiation of HCC [3, 4]. As a multifunctional oncoprotein, HBx localizes in the cytoplasm, nucleus, and mitochondria, where it affects signal transduction, transcription, and mitochondrial function [5, 6].

NLR pyrin domain containing 3 (NLRP3) is a cytoplasmic pattern recognition receptor that is widely distributed in hepatic parenchymal cells and non-substantial cells [7–9]. The NLRP3 inflammasome, which consists of NLRP3, inflammasome adaptor protein apoptosis-associated speck-like protein containing CARD (ASC), and pro-caspase-1, requires two signals to be activated. The initiation signal is mediated by nuclear factor (NF)-κB, which upregulates expression of the inflammasome-related proteins; while, the second signal is mediated by endogenous or exogenous hazard signals [10–12]. The activation of NLRP3 promotes the production of active caspase-1, which contains two heterodimers of p20 and p10. This activation then induces the maturation and secretion of inflammatory cytokines, namely interleukin (IL)-1β, IL-18, and high-mobility group box 1 protein (HMGB1), as well as the induction of inflammatory necrosis (pyroptosis) [13–16]. Increasing evidence indicates that the inflammasome is involved in various liver diseases, including liver injury, hepatitis, liver fibrosis, and cirrhosis; however, whether the NLRP3 inflammasome participates in HBx-induced hepatitis remains unclear.

The mitochondrial ROS (mitoROS) model is a widely accepted mechanistic explanation for NLRP3 activation [11, 17]. Physiological levels of ROS maintain normal cell signaling and homeostasis; however, abnormally high levels of ROS activate several signaling molecules, including NF-κB, mitogen-activated protein kinases (MAPKs), protein kinase B (Akt), and signal transducer and activator of transcription 3 (STAT3), resulting in cellular inflammation and apoptosis [18]. Given that the mitochondrial oxidative respiratory chain serves as the primary source of intracellular ROS and that the liver is a mitochondria-rich organ, it is plausible that the mitoROS model may contribute significantly to the development and progression of liver diseases. Further, our previous studies showed that HBx interacts with cytochrome c oxidase subunit 3 (COXIII), a protein related to mitochondrial respiratory chains, and causes an increase in mitoROS levels, resulting in decreased membrane potential, ATP synthesis disorder, and cytosolic calcium overload, ultimately causing mitochondrial dysfunction [19, 20].

In the current work, we sought to investigate whether HBx promoted mitoROS-mediated liver inflammatory injury via activation of the NLRP3 inflammasome. We also examined the role of HBx in hepatocyte pyroptosis under oxidative stress.

## Materials and methods

### Patient tissue and serum samples

Archived paraffin-embedded HCC tissues and matched non-tumor tissues collected from 51 patients from 2014 to 2017 at Union Hospital of Fujian Medical University, China, were randomly selected. Written informed consent was obtained before surgical resection. Additionally, 84 serum samples, including 23 HBV-negative and 61 HBV-positive samples from patients collected between 2017 and 2018, were evaluated (tissue and serum samples were from different subjects). The inclusion criterion was patients who were first diagnosed with HBV infection and did not receive antiviral therapy. The exclusion criterion were patients with other hepatitis virus infections, nonviral hepatitis (alcoholic or non-alcoholic hepatitis, drug-induced hepatitis, etc.), and autoimmune hepatitis. All clinical samples were collected according to protocols approved by the Medical Faculty of Fujian Medical University Ethics Committee (Approval number 2019Y001).

### Cell culture and plasmids

Normal human liver HL7702 cells (Shanghai Cell Biology Institute of Chinese Academy of Science, Shanghai, China) were cultured in Roswell Park Memorial Institute (RPMI) medium supplemented with 10% fetal bovine serum (Hyclone, Logan, UT, USA). For induction of oxidative stress, cells were treated with 100-μM hydrogen peroxide (H_2_O_2_) for 12 h after 36-h plasmid transfection. The other groups that were transfected with plasmids for 48 h, however, did not undergo induced oxidative stress. The pHBx plasmid expressing HBx and pcDNA3.1 (pNC) was maintained in our laboratory. The pGEM-4Z (pGEM) plasmid was purchased from Promega (#P2161; Madison, WI, USA). The pHBV plasmid expressing full-length wild-type HBV genomic complementary DNA (cDNA) and pHBV-HBx-expressing cDNA of an HBx-deficient HBV mutant were gifted from Professor M.J. Bouchard (Drexel University, Philadelphia, PA, USA) [21, 22]. Plasmid transfections were performed using Lipofectamine 3000 (#L3000-008; Invitrogen, Carlsbad, CA, USA). *N*-acetyl-l-cysteine (NAC; #A7250; Sigma-Aldrich, St. Louis, MO, USA) was dissolved in distilled deionized water (ddH_2_O) and added to cells at a final concentration of 5 nM prior to H_2_O_2_ treatment for 60 min. Mito-TEMPO (#SML0737; Sigma-Aldrich, St. Louis, MO, USA) was dissolved in ddH_2_O and added to cells at a final concentration of 50 μM before H_2_O_2_ treatment for 60 min.

### Cell viability

Cell viability was determined using a Cell Counting Kit-8 (CCK-8; #CK04-11; Dojindo, Kumamoto, Japan) as described by the manufacturer. Briefly, HL7702 cells were plated into 96-well plates and grown to 70–80% confluence. The cells were then treated with different H_2_O_2_ concentrations (0, 25, 50, 75, 100, 125, 150, 175, and 200 μM). After 12 h of H_2_O_2_ treatment, 10-µl CCK-8 reagent was added to each well and incubated for 1 h at 37 °C. The absorbance of each well was read at 450 nm using a spectrophotometric reader.

### Enzyme-linked immunosorbent assay (ELISA)

The levels of ASC, IL-1β, IL-18, and HMGB1 in cellular medium were detected by double-antibody sandwich ELISA using Quantikine ELISA kits (R&D Systems, Minneapolis, MN, USA) according to the manufacturer’s instructions. The optical density (OD) at 450 nm for each well was determined using a plate reader (Bio-Rad, Hercules, CA, USA).

### Quantitative reverse transcription-polymerase chain reaction (qRT-PCR)

Total RNA was extracted using TRIzol Reagent (#15596026; Invitrogen, Carlsbad, CA, USA). Following reverse transcription, the quantitative PCR reactions were performed using SYBR Green PCR Master Mix (#17747200; Roche, Basel, Switzerland) according to the manufacturer’s protocol. The primer sequences are provided in Table 1. The data from five independent experiments were analyzed using the 2^−∆∆*C*t^ method values.

**Table 1:** Primers used for RT-PCR

Gene	Primer sequences
NLRP3	Forward 5′-GGTGGAGTGTCGGAGAAG-3′
	Reverse 5′-CTGTCATTGTCCTGGTGTCT-3′
ASC	Forward 5′-GCTGCTGGATGCTCTGTA-3′
	Reverse 5′-AGGCTGGTGTGAAACTGAA-3′
Caspase-1	Forward 5′-GAGCAGCCAGATGGTAGAG-3′
	Reverse 5′-CCCACAGACATTCATACAGTTTC-3′
IL-1β	Forward 5′-TCACCTCTCCTACTCACT-3′
	Reverse 5′-CGGTTGCTCATCAGAATG-3′
IL-18	Forward 5′-GACCTTCCAGATCGCTTCCTC-3′
	Reverse 5′-GATGCAATTGTCTTCTACTGGTTC-3′
HMGB1	Forward 5′-TCAAAGGAGAACATCCTGGCCTGT-3′
	Reverse 5′-CTGCTTGTCATCTGCAGCAGTGTT-3′
GSDMD	Forward 5′-AGACCATCTCCAAGGAACTG-3′
	Reverse 5′-GGACAACACCAGGCACTC-3′
GAPDH	Forward 5′-GAAGGTGAAGGTCGGAGTC-3′
	Reverse 5′-GAAGATGGTGATGGGATTTC-3′

*NLRP3* NLR pyrin domain containing 3, *ASC* apoptosis-associated speck-like protein containing a caspase recruitment domain (CARD), *HMGB1*, high-mobility group box 1, *GSDMD* gasdermin D, *GAPDH* glyceraldehyde-3-phosphate dehydrogenase

### Western blotting

Protein expression was evaluated by western blotting, as described previously [23]. Antibodies specific for NF-κB p65 (#8242; Cell Signaling Technology, Danvers, MA, USA), NLRP3 (#sc-66846; Santa Cruz Biotechnology, Santa Cruz, CA, USA), pro-caspase-1 (#IMG-5028; Novus Biologicals, Littleton, CO, USA), caspase-1 (#sc-56036; Santa Cruz Biotechnology, Santa Cruz, CA, USA), IL-1β (#ab9722; Abcam, Cambridge, MA, USA), glyceraldehyde-3-phosphate dehydrogenase (GAPDH) (#2118; Cell Signaling Technology, Danvers, MA, USA), ASC (#ab180799; Abcam, Cambridge, MA, USA), gasdermin D (GSDMD) (#ab210070; Abcam, Cambridge, MA, USA), HBx (#ab39716; Abcam, Cambridge, MA, USA), hepatitis B virus core protein (HBc) (#MAB16989; Merck Millipore, Billerica, MA, USA), and cytochrome c oxidase subunit 4 (COXIV) (#GB11250; Wuhan Goodbio Technology, Wuhan, China) were used.

### Immunofluorescence

HL7702 cells were plated into 24-well plates and grown to 50% confluence. Following plasmid transfection and H_2_O_2_ induction, immunofluorescence was performed. Briefly, cells were fixed with 4% paraformaldehyde for 15 min and permeabilized with 0.25% Triton X-100 for 15 min. Cells were then blocked with 10% donkey serum for 60 min, incubated with primary antibodies overnight at 4 °C, and immunostained with secondary antibodies for 60 min at 23–25 °C. Primary antibodies specific for ASC (#ab180799; Abcam, Cambridge, MA, USA) and HBx (#ab235; Abcam, Cambridge, MA, USA) were used. The secondary donkey anti-mouse IgG (H + L), Alexa Fluor 488 (#A-21202; Invitrogen, Carlsbad, CA, USA), donkey anti-rabbit IgG (H + L), and Alexa Fluor 555 (#A-31572; Invitrogen, Carlsbad, CA, USA) antibodies were used. Finally, cells were observed using fluorescence confocal microscopy (Zeiss FM780, Jena, Germany). Pictures were taken under 63 × oil microscope and excitation light wavelengths of 405 nm, 488 nm, and 594 nm were selected for layer scanning with a scanning interval of 0.35 um/layer. The fluorescence excitation displayed blue, green, and red, respectively.

### Mitochondria isolation

Mitochondrial proteins were isolated according to the introduction of cellular mitochondria isolation kit (#89874; Thermo Fisher Scientific, Waltham, MA, USA). Briefly, cells with 100-μM H_2_O_2_ treating for 12 h after 36-h plasmid transfection were firstly harvested with trypsin and homogenized with Dounce Tissue Grinder on ice. Then, the post-nuclear fractions were obtained by centrifuging at 700×*g* for 10 min at 4 °C. To obtain a more purified fraction of mitochondria, the post-nuclear fractions were centrifuged at 3000×*g* for 15 min. Finally, the mitochondria pellet was dissociated with lysis buffer (#C3601-4; Beyotime Biotechnology, Shanghai, China) and then subjected to western blotting analysis.

### Lactate dehydrogenase (LDH) release assay

HL7702 cells were cultured in 96-well plates at a density of 5000 cells/well. After transfection with plasmids and H_2_O_2_ induction, the culture medium was collected. The release of lactate dehydrogenase (LDH) was determined using an LDH Cytotoxicity Detection Kit (#04744926001; Roche, Basel, Switzerland) according to the manufacturer’s protocol. The OD at 490 nm for each sample was determined using a microplate reader (Bio-Rad, Hercules, CA, USA).

### Propidium iodide (PI) staining

Membrane pore formation was detected by PI and Hoechst 33342 staining (#C1056; Beyotime Biotechnology, Shanghai, China). HL7702 cells were seeded into 6-well plates, transfected with plasmids, and exposed to H_2_O_2_. After treatment, the cells were washed with phosphate-buffered saline (PBS) and stained with Hoechst 33342 (5 µl) and PI (5 µl) for 20 min at 37 °C. The cells were washed with PBS three times and observed using a fluorescence microscope (TE2000-U, NIKON, Tokyo, Japan). The percentage of PI-positive cells was determined in three random microscopic fields.

### Transmission electron microscopy (TEM)

Cell membrane integrity and mitochondrial morphological changes were determined using TEM. Cells were collected and fixed overnight at 4 °C in 0.1-M sodium cacodylate solution (pH 7.3) containing 2% fresh glutaraldehyde. The fixed cells were treated with 1% osmium tetroxide in 0.1-M cacodylate solution at 4 °C for 1 h and then stained with 1% uranyl acetate, dehydrated with ethanol, and embedded in epoxy resin. Finally, 90-nm ultrathin sections were cut and post-stained with uranyl acetate and bismuth subnitrate. The samples were visualized by TEM (TEC-NAI 1200, FEI Italia, Milan, Italy).

### Measurement of mitochondrial ROS levels

Mitochondrial levels of ROS were determined using MitoSOX™ Red (#M36008; Molecular Probes, Eugene, OR, USA) according to the manufacture’s protocol. Cells in 6-well plates were transfected and treated with or without 100 μM H_2_O_2_. After treatment, the culture medium was removed, and the trypsinized cells were treated with 5 µM MitoSOX™ Red reagent at 37 °C for 10 min. Cells were then washed with warm PBS thrice. Fluorescence intensity was determined using BD Accuri™ C6 Flow Cytometer (BD Biosciences, San Jose CA, USA). The FlowJo software v7.6 was used to measure the mean fluorescence intensity.

### Immunohistochemistry

Immunohistochemistry was performed using Polink-2 plus Polymer HRP Detection System (#PV9001 and PV9003; Zhongshan Golden Bridge Biotechnology, Beijing, China) according to the manufacture’s protocol. Primary antibodies specific for NLRP3 (#ab4207; Abcam, Cambridge, MA, USA), ASC (#ab180799; Abcam, Cambridge, MA, USA), and IL-1β (#ab9722; Abcam, Cambridge, MA, USA) were incubated with sections overnight at 4 °C. Representative images of 10 random fields per section were obtained microscopically (DMil, Leica, Wetzlar, Germany). The mean integrated optical density (IOD) was determined using Image-Pro Plus software v6.0 (Media Cybernetics, Rockville, MD, USA) to evaluate the expression levels of proteins.

### Statistical analysis

Data were assessed to evaluate the normal distribution and homogeneity of variance by the Shapiro–Wilk normality test and Levene’s test, respectively. Comparisons between two groups following a normal distribution and equal variance were performed using a two-tailed Student’s *t* test; whereas, one-way analysis of variance (ANOVA) with Tukey post-test was used for multiple comparisons. For a non-normal distribution or uneven variance, the Wilcoxon rank-sum and Kruskal–Wallis test with Dunnett’s post hoc test were performed to compare two groups or multiple groups. Data are shown as mean values ± standard deviation (SD). *P* < 0.05 was considered statistically significant. All data were analyzed using SPSS software v13.0.1 (SPSS, Inc., Chicago, IL, USA).

## Results

### Optimization of H_2_O_2_-intervention concentration and verification of recombinant plasmid expression in HL7702 cells

**Fig. 1:**
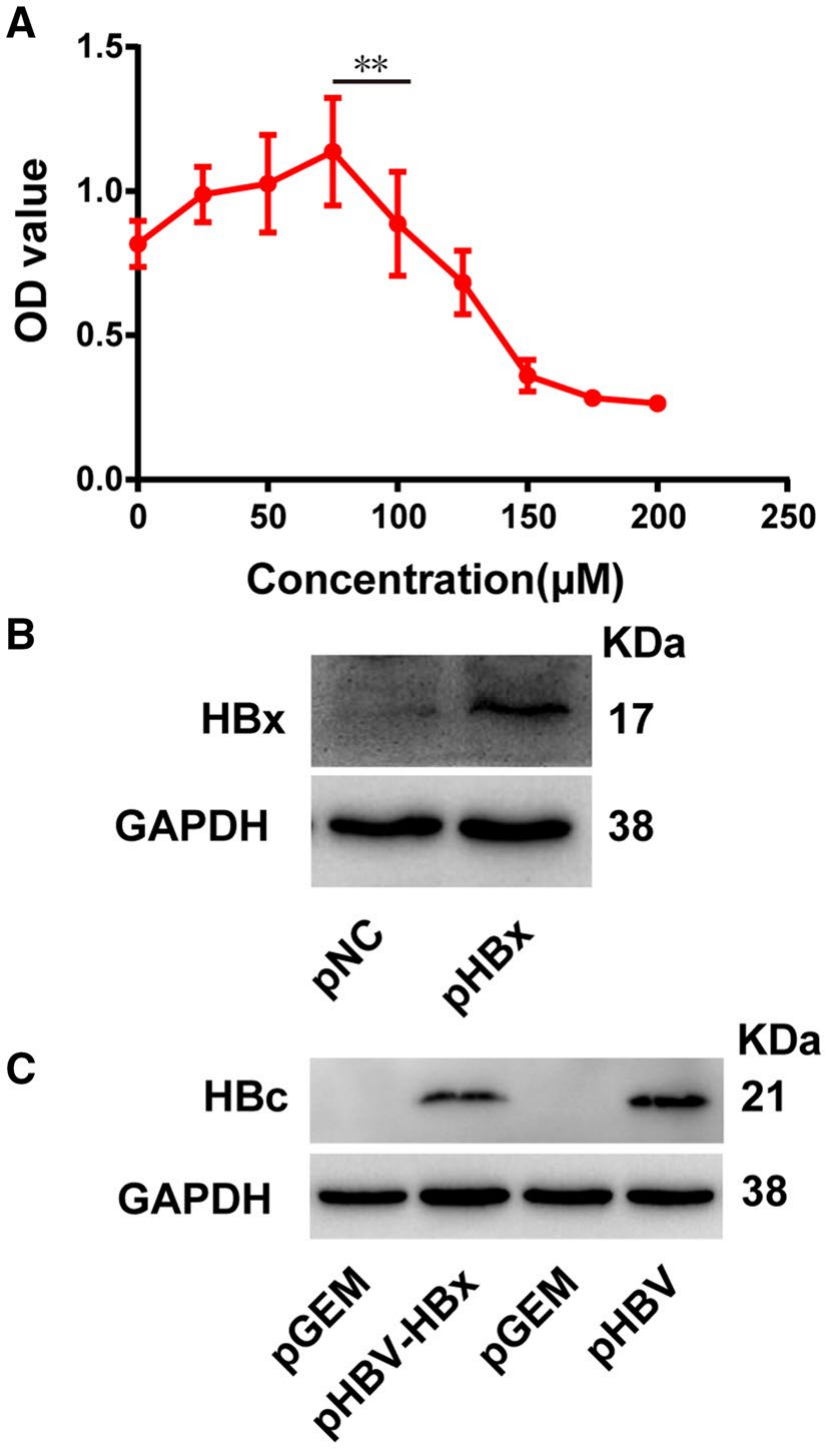
Cytotoxicity of H_2_O_2_ and verification of recombinant plasmid expression in HL7702 cells. Cells were treated with different concentrations of H_2_O_2_, as indicated for 12 h. Cell viability was evaluated using a Cell Counting Kit-8 assay, *n* = 4 (**a**). HBV X antigen (HBx) and C antigen (HBc) were transiently expressed in HL7702 cells and protein expression levels determined by western blotting, *n* = 4 (**b**, **c**). Abbreviations: pNC, cells transfected with control vector pcDNA3.1; pHBx, cells transfected with reconstituted pHBx vector; pGEM, cells transfected with control vector pGEM-4Z; pHBV-HBx, cells transfected with pHBV-HBx vector; pHBV, cells transfected with pHBV vector. Data are displayed as mean ± SD. *P < 0.05 and **P < 0.01

To determine the optimal concentration for H_2_O_2_ intervention, different concentrations of H_2_O_2_ were applied to hepatocytes to induce oxidative stress. As indicated by decreased cell viability, cells incurred oxidative-stress-induced damage from H_2_O_2_ treatment for 12 h at a concentration of 100 μM (Fig. 1a). Accordingly, this treatment was used in subsequent experiments as the moderate exposure model. To assess protein expression of transfected plasmids in HL7702 cells, western blotting was performed. The results showed that pHBx, pHBx-HBx, and pHBV successfully expressed their corresponding proteins; while, no expression was detected from the control plasmids (Fig. 1b, c).

### HBx induces a pro-inflammatory response under H_2_O_2_ stimulation in hepatocytes

**Fig. 2:**
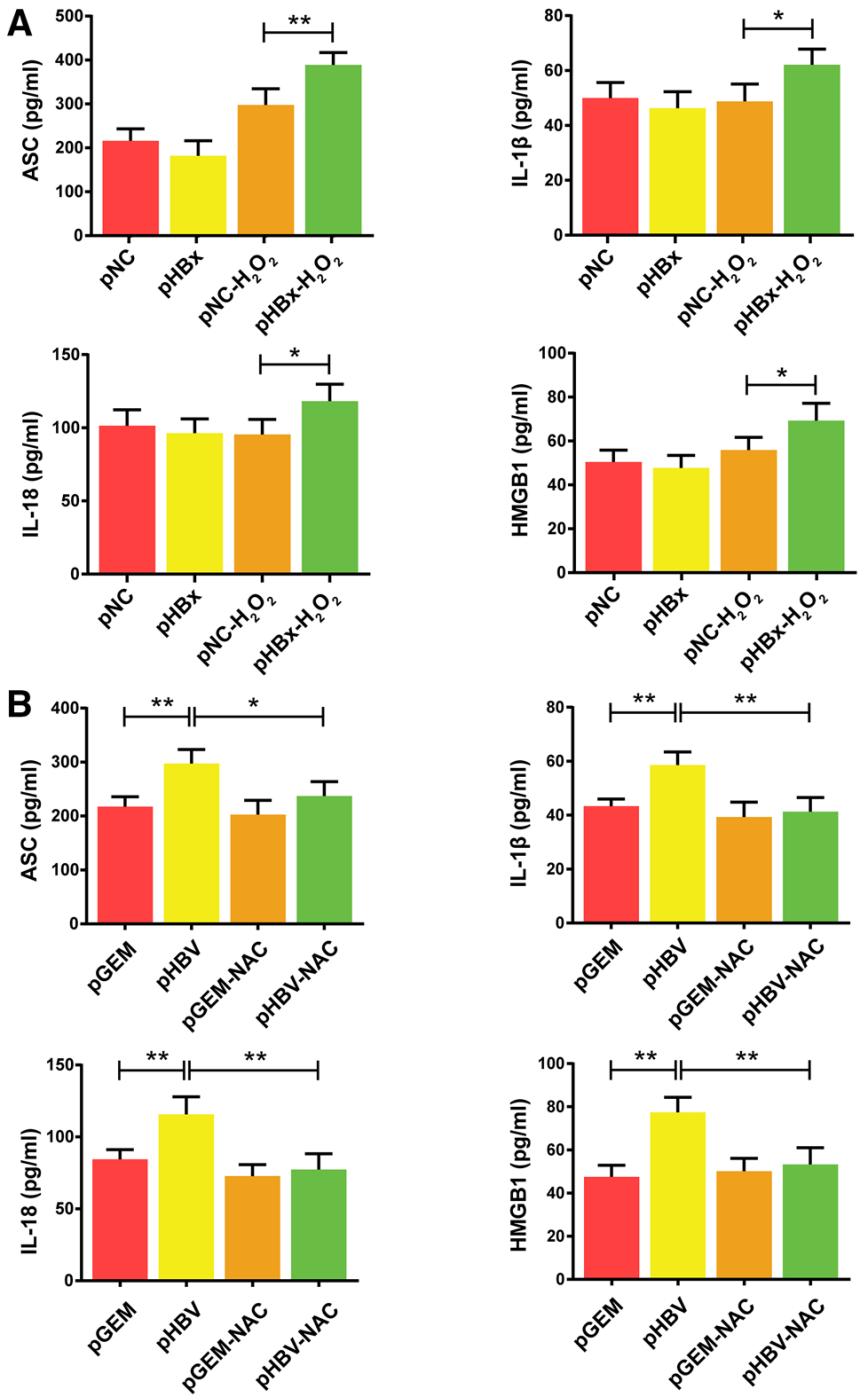
Impact of hepatitis B virus (HBV) X protein (HBx) on the expression of inflammatory mediators in hepatocytes. Concentrations of ASC, IL-1β, IL-18, and HMGB1 in the culture media of HBx-expressing HL7702 cells with or without H_2_O_2_ stimulation (100 μM) were determined by ELISA, *n* = 4 (**a**). Concentrations of ASC, IL-1β, IL-18, and HMGB1 from HBV-expressing HL7702 cells were quantified by ELISA, *n* = 4 (**b**). Data are presented as mean ± SD. *P < 0.05 and **P < 0.01

Previous reports suggest that NLRP3 inflammasome-associated pro-inflammatory factor levels increase in several inflammatory liver diseases [24, 25]. To determine whether HBx promoted the secretion of inflammatory cytokines in normal liver cells, HL7702 cells were transfected with HBx and HBV-expression plasmids. Under H_2_O_2_ stress, the release of ASC, IL-1β, IL-18, and HMGB1 into the culture medium increased in HBx-transfected cells (Fig. 2a). To explore whether HBV replication without H_2_O_2_ stress-induced similar results, HBV-expressing and control plasmids were transfected into HL7702 cells. Similar to that observed in the HBx-expressing cells exposed to H_2_O_2_, full-length HBV-expression stimulated secretion of inflammatory cytokines into the cell culture medium (Fig. 2b). Collectively, these results suggest that HBx enhanced the release of ASC, IL-1β, IL-18, and HMGB1 from normal hepatocytes in vitro in an oxidative stress environment.

#### HBx-induced NLRP3 inflammasome upregulation and activation under oxidative stress

**Fig. 3:**
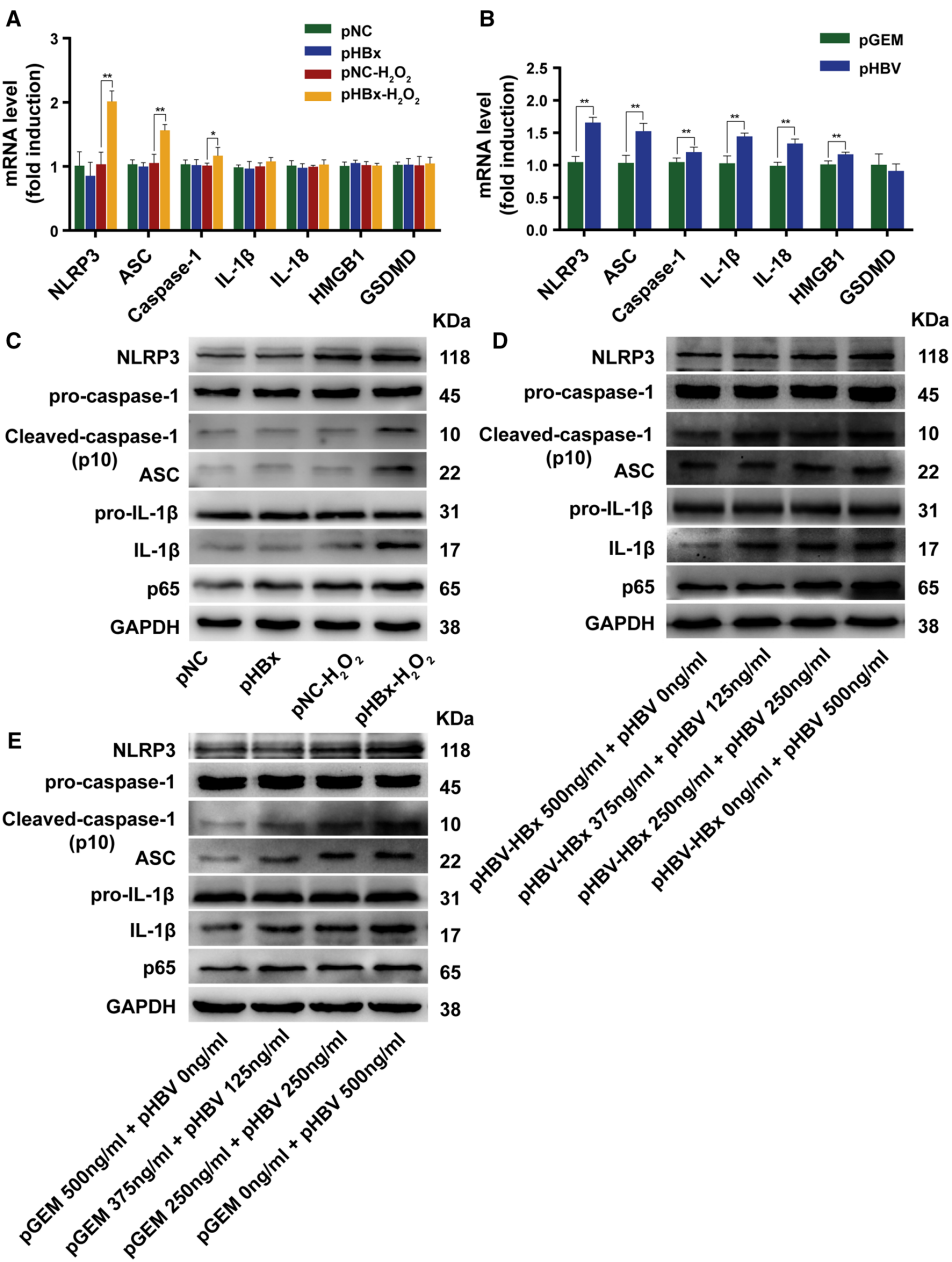
HBV X protein (HBx) promoted the upregulation and activation of NLRP3 inflammasome expression under H_2_O_2_ stress. Quantification of mRNA expression of GAPDH, NLRP3, ASC, caspase-1, IL-1β, IL-18, HMGB1, and GSDMD using real-time PCR, *n* = 5 (**a**, **b**). Data are expressed as mean ± SD. *P < 0.05 and **P < 0.01. Western blot analysis of NLRP3, ASC, pro-caspase-1, Cleaved-caspase-1 (p10), and IL-1β (mature IL-1β), *n* = 4 (**c**, **d**). GAPDH served as a loading control

Since activation of the NLRP3 inflammasome promotes the transformation of pro-caspase-1 into its active p20 and p10 heterodimers as well as the production of mature IL-1β, IL-18, and HMGB1, we next determined whether the oncoprotein HBx impacted the NLRP3 inflammasome in an oxidative stress microenvironment. To this end, HL7702 cells were transfected with pNC or pHBx and stimulated with H_2_O_2_. HBx-expressing hepatocytes demonstrated enhanced mRNA levels of NLRP3, ASC, and caspase-1 only in an H_2_O_2_-stress microenvironment (Fig. 3a). However, HBV-expression cells demonstrated elevated NLRP3, ASC, caspase-1, IL-1β, IL-18, and HMGB1 in the absence of H_2_O_2_ treatment (Fig. 3b). Subsequently, protein levels of NLRP3-related molecules were detected. First, higher expression of NLRP3, Cleaved-caspase-1 (p10), ASC, IL-1β, and NF-κB p65 was observed only in the HBx cells exposed to H_2_O_2_, and not in HBx cells without H_2_O_2_ intervention (Fig. 3c). Next, in an environment of HBV replication without H_2_O_2_ treatment, we determined that HBx also promoted the expression of NLRP3-related proteins in a dose-dependent manner (Fig. 3d). Furthermore, the expression of NLRP3-related proteins was similarly upregulated by HBV (Fig. 3e). Although the total pro-caspase-1 and pro-IL-1β protein levels did not change, the increased levels of Cleaved-caspase-1 (p10) and mature IL-1β suggested that the NLRP3 inflammasome was activated.

#### HBx-induced pyroptosis in HL7702 cells treated with H_2_O_2_

**Fig. 4:**
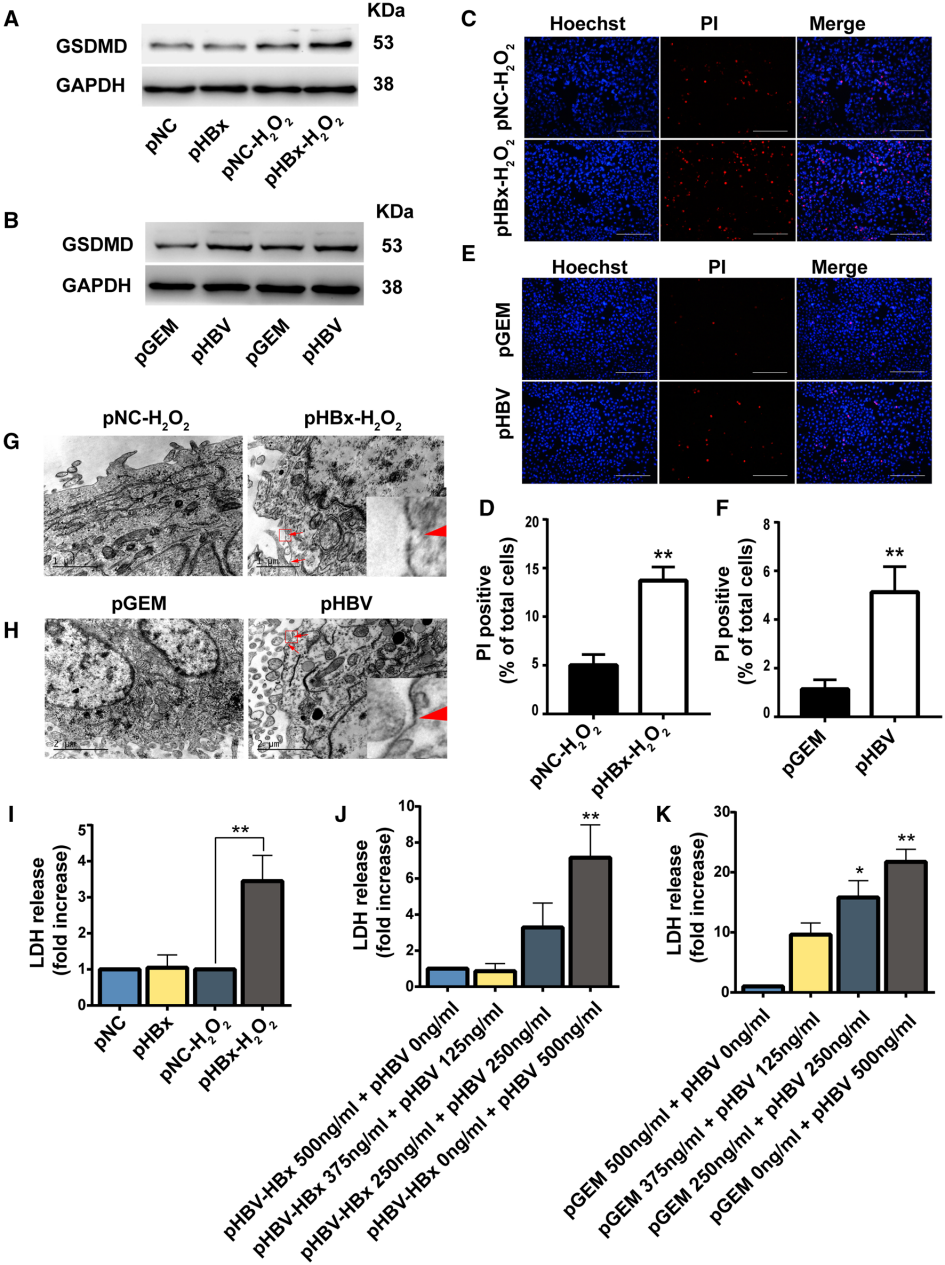
Hepatitis B virus X protein (HBx) induced liver cell pyroptosis under H_2_O_2_ stimulation. Western blot analysis of GSDMD and loading control GAPDH, *n *= 4 (**a**, **b**). Representative micrographs of PI staining (Red) and Hoechst 33342 staining (Blue), *n* = 5 (**c**–**f**). Scale bar indicates 200 µm. The pore formation of the cellular membrane was examined by TEM, *n* = 4 (**g**, **h**). Levels of LDH released into the cell culture medium from membrane pores were examined using an LDH Cytotoxicity Detection Kit, *n* = 5 (**i**–**k**). Data are expressed as mean ± SD. *P < 0.05 and **P < 0.01

Since activation of the inflammasome triggered pyroptosis, we next investigated whether HBx would induce pyroptosis in cells exposed to oxidative stress. Pyroptosis is dependent upon active caspase-1, GSDMD, membrane pore formation, and cytolysis, resulting in leakage of LDH [9, 26]. Thus, we quantified the expression level of GSDMD, pore formation, and LDH leakage. The results revealed that HBx increased GSDMD expression in cells exposed to H_2_O_2_; whereas, cells not under H_2_O_2_-stimulated conditions failed to show a difference (Fig. 4a). Similarly, we observed a higher expression of GSDMD in HBV-expressing cells without H_2_O_2_ stress compared with the control group (Fig. 4b). Pore formation was detected using PI staining and TEM. PI staining revealed that HBx significantly increased PI uptake in cells treated with H_2_O_2_ (Fig. 4c, d). Consistent with these findings, we also observed a higher number of PI-positive cells in HBV-expressing cell cultures (Fig. 4e, f). Moreover, TEM revealed membrane pore formation in HBx cells exposed to H_2_O_2_ and HBV cells without H_2_O_2_ treatment (Fig. 4g, h). HBx-induced pyroptosis was further confirmed by increased LDH levels in cell culture medium (Fig. 4i–k).

#### Mitochondrial ROS mediated HBx-induced NLRP3 activation and pyroptosis in liver cells

**Fig. 5:**
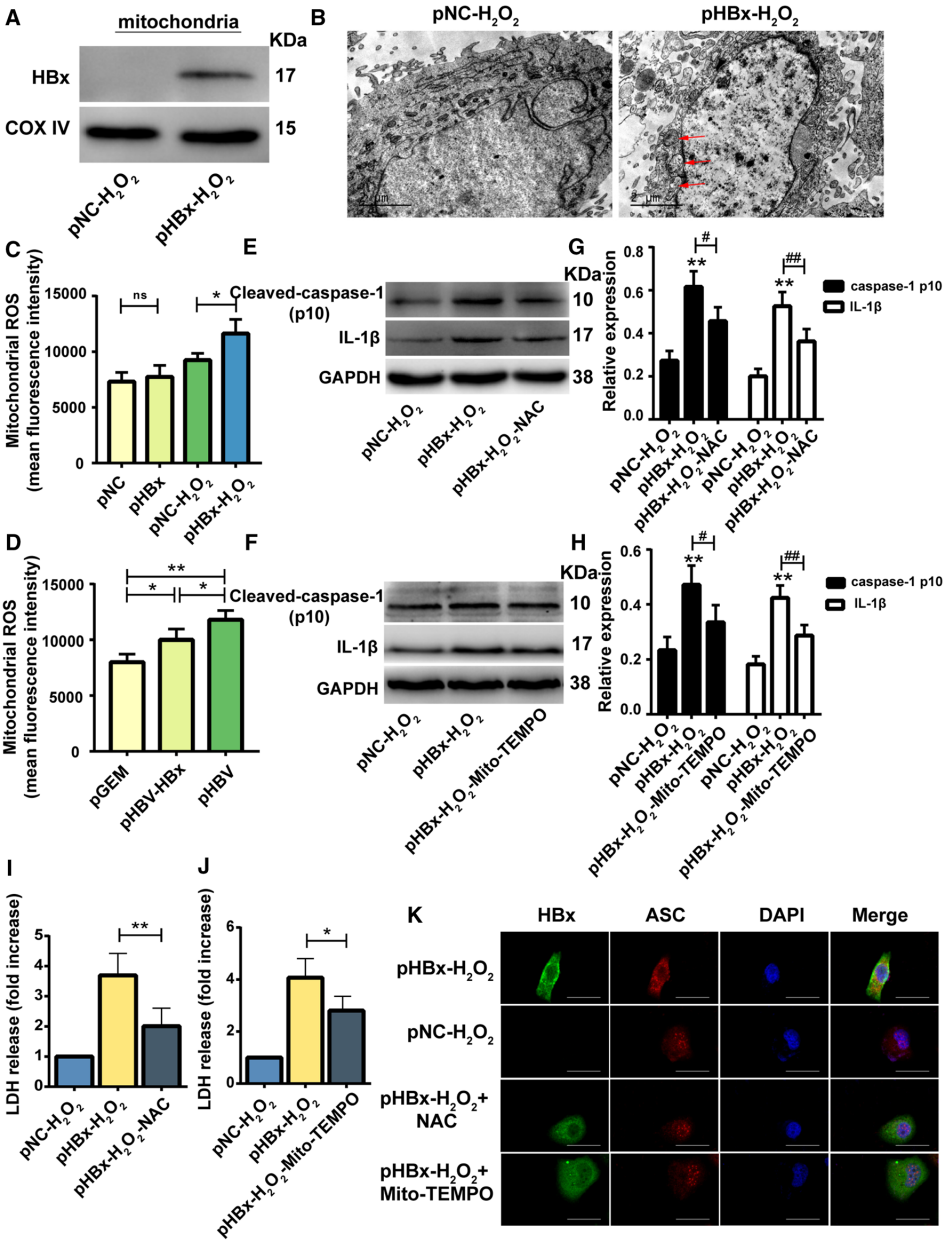
Mitochondrial reactive oxygen species (mitoROS) mediated HBV X protein (HBx)-induced NLRP3 activation and pyroptosis in liver cells. Western blot analysis of HBx localized in the mitochondria. COXIV served as a loading control of mitochondrial protein, *n* = 4 (**a**). Morphological changes of mitochondria were observed by TEM, *n* = 4 (**b**). mitoROS levels were examined by flow cytometry, *n* = 4 (**c**, **d**). Effects of the ROS scavenger NAC and specific mitoROS inhibitor mito-TEMPO on HBx-induced inflammasome activation analyzed by western blotting, *n* = 4 (**e**–**h**). NAC and mito-TEMPO attenuated HBx-induced LDH release, *n* = 5 (**i**, **j**). NAC and mito-TEMPO inhibited both the translocation of ASC and formation of ASC specks, as detected by immunofluorescence, *n* = 4 (**k**). Data are shown as mean ± SD. *P < 0.05, **P < 0.01,#*P *< 0.05, ##*P *< 0.01

Mitochondrial damage that causes increased intracellular ROS levels promotes inflammasome activation and pyroptosis [27]. We, therefore, investigated whether mitoROS was involved in the HBx-induced NLRP3 activation and pyroptosis upon H2O2 stress. To verify the mitochondrial localization of HBx, the mitochondrial protein was extracted and tested for HBx. Mitochondria of HBx-expressing hepatocytes were found to contain HBx protein (Fig. 5a). TEM results revealed overt mitochondria swelling in HBx-expressing HL7702 cells with H2O2 treatment compared with that of the control group (Fig. 5b). Additionally, H2O2-exposed HBx-expressing cells had elevated levels of mitoROS, as compared with those of H2O2-exposed cells not expressing HBx (Fig. 5c). Cells transfected with pHBV-HBx or pHBV promoted increased levels of mitoROS, compared with those of the pGEM group (Fig. 5d). Also, the pHBV group had higher mitoROS levels, as compared with those of the pHBV-HBx group. Further, HBx-induced activation of the inflammasome was partially abrogated following treatment with the ROS scavenger NAC and specific mitoROS inhibitor mito-TEMPO, as suggested by the reduction of Cleaved-caspase-1 (p10) and IL-1β expression, PI positivity, LDH release, and inflammatory cytokine secretion (Figs. 5e–j, 2b, Supplementary Fig. 1a–b). Immunofluorescence imaging demonstrated higher expression of ASC in HBx cells under H2O2 stress, compared with that of the control group; while, it was also apparent that HBx promoted the translocation of ASC from the nucleus to the cytoplasm as well as the formation of ASC specks in the cytoplasm (Fig. 5k), thereby further confirming activation of the NLRP3 inflammasome. Moreover, the translocation of ASC was partially inhibited by treating cells with the ROS scavenger NAC, and mito-TEMPO.

### Association between the expression level of NLRP3 inflammasome components and HBV DNA load

**Fig. 6:**
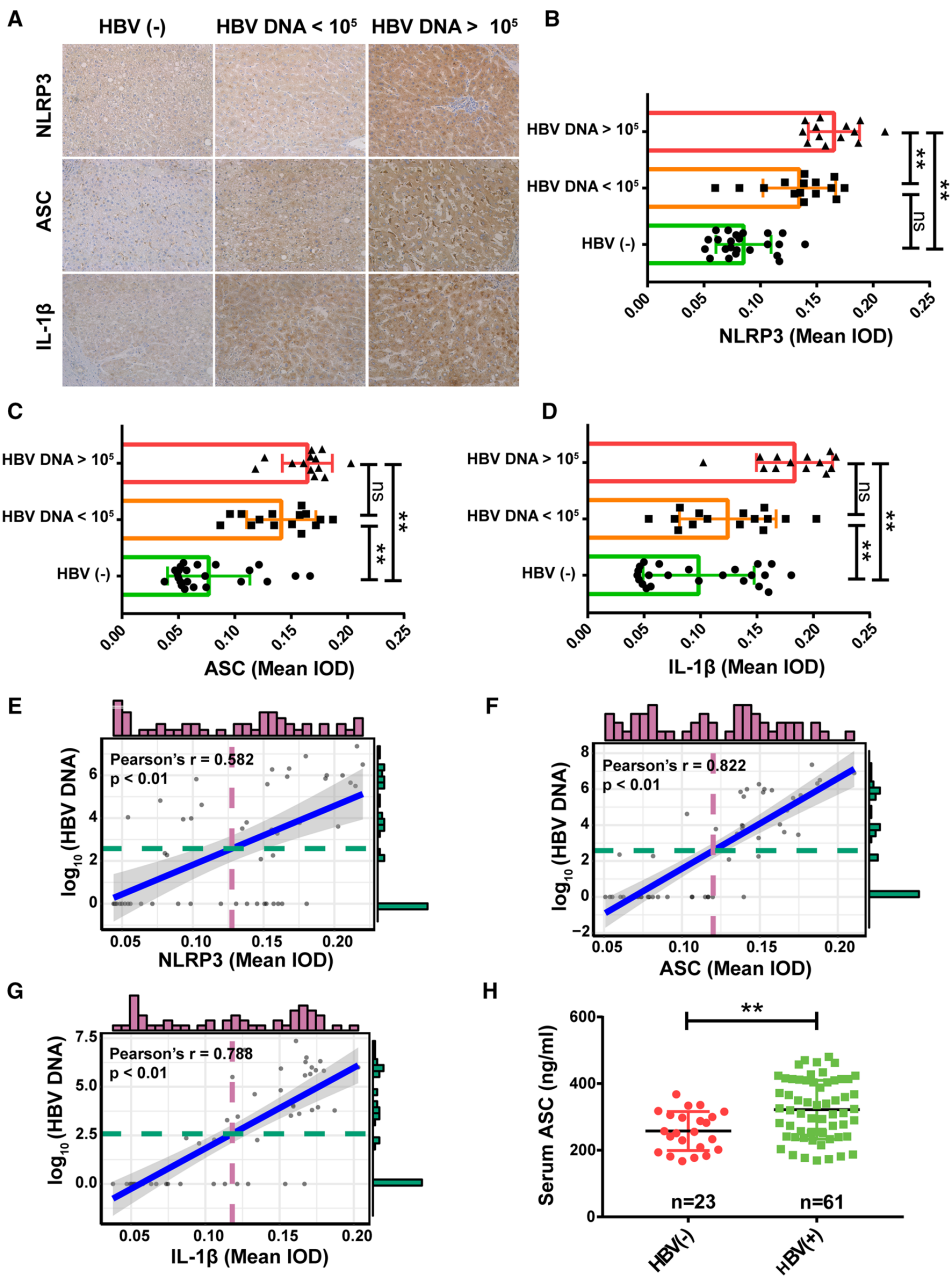
Association between the expression levels of NLRP3 inflammasome components and hepatitis B virus (HBV) DNA load. Analysis by immunohistochemistry of expression levels of NLRP3, ASC, and IL-1β in tumor-adjacent tissues of HCC patients with or without HBV infection. Representative images are shown, and the mean integrated optical density (IOD) of protein expression was statistically analyzed, *n* = 51 (**a**–**d**). Pearson correlation analysis of NLRP3, ASC, and IL-1β expression and HBV DNA copy number in a cohort of 51 patients, *n* = 51 (**e**–**g**). Serum ASC in patients with varied HBV DNA load was detected by ELISA, *n* = 84 (**h**). The height of the histogram represents the DNA copy number counts. Data are shown as mean ± SD. *P < 0.05 and **P < 0.01

To explore the clinical significance of HBV-induced changes in the expression of NLRP3 inflammasome components, a total of 51 HBV-involved HCC adjacent tissues were evaluated. Immunohistochemistry results showed that cytoplasmic levels of NLRP3, ASC, and IL-1β were weakly present in HBV-negative patients; however, a much stronger signal was detected in HBV-positive patients. Moreover, patients with a higher HBV DNA copy number (> 10^5^ IU/ml) had the highest cytoplasmic staining of NLRP3 (Fig. 6a–d). Correlation analysis further demonstrated that the expression level of these proteins positively correlated with HBV DNA titers (Fig. 6e–g).

Next, given that no previous research has detected the expression of serum ASC in HBV patients, we evaluated ASC concentrations in 84 serum samples from patients with or without HBV infection. In accordance with the result of ASC content in the cell culture medium (Fig. 2a–b) and cytoplasmic staining of ASC in tissues (Fig. 5a), HBV-positive patients exhibited higher ASC concentrations in their sera compared to those of HBV-negative patients (Fig. 6h). These results strongly suggest that the NLRP3 inflammasome pathway is involved in HBV-related hepatitis.

## Discussion

Inflammation is the hallmark of acute and chronic liver diseases. Persistent chronic inflammation can lead to liver fibrosis, cirrhosis, and even liver cancer [28]. Among HBV proteins, HBx is a leading mediator of hepatic inflammation [29, 30]. Considering that early HBV infection is primarily characterized by chronic liver inflammation, rather than employ a model of liver cancer development, we instead chose to use the standard liver cell line HL7702 to explore activation of intrahepatic cytokine networks induced by HBx. In the current study, we found that under oxidative stress, HBx enhanced NLRP3 inflammasome-mediated inflammation and pyroptosis through upregulating mitoROS production. Interestingly, in a setting of HBV replication without H_2_O_2_ stress, HBx played a similar role in mediating the NLRP3-associated inflammatory process. To the best of our knowledge, this study provides the first evidence that HBx-mediated activation of the NLRP3 inflammasome and pyroptosis required conditions, such as oxidative stress or HBV replication.

Oxidative stress is a prominent indicator of HBV infection. It is characterized by increased oxidative products, reduced antioxidant enzymes, and elevated ROS levels, including those of H_2_O_2_ [31–33]. Similarly, we found that HBx enhanced the levels of malondialdehyde, a product of fatty acid oxidation, and reduced the levels of the antioxidant superoxide dismutase in mice (data not shown). However, our previous studies demonstrated that HBx alone is not sufficient to induce oxidative stress [20]. Therefore, in this study, we used H_2_O_2_ to induce oxidative stress in cells prior to evaluating the effect of HBx on the NLRP3 inflammasome, to simulate the intrahepatic oxidative stress environment. We found that HBx-expressing cells promoted the secretion of ASC, IL-1β, IL-18, and HMGB1 under H_2_O_2_ stress. Also, HBV-expressing cells without H_2_O_2_ intervention also promoted the release of inflammatory cytokines. Hence, other HBV proteins may induce oxidative stress after which HBx may promote NLRP3 activation. The observed ROS levels between the pGEM and pHBV-HBx groups (Fig. 5d) confirmed this hypothesis. We also observed opposing roles for HBx on apoptosis. Similarly, Huang et al. found that HBx suppresses apoptosis of hepatoma cells during starvation by enhancing mitophagy; however, it exhibits the opposite role under well-fed conditions [23]. Taken together, these results suggest that HBx may exert specific and unique roles in different environments, while also stimulating the release of pro-inflammatory cytokines only in the presence of other specific factors, such as oxidative stress, nutrient deprivation, or the expression of other HBV proteins.

Mitochondrial injury often leads to the generation of ROS and subsequently activates the ROS signaling pathway. Rahmani et al. showed that HBx interacts with the mitochondrial human voltage-dependent anion-selective channel protein 3 (HVDAC3) and alters mitochondrial membrane potential [34]. Consistent with this, we found that HBx was present in the mitochondrial fraction isolated from HBx-expressing cells. TEM results demonstrated morphological changes consistent with mitochondrial damage. Moreover, exposure to H_2_O_2_ caused an increase in mitoROS levels in HBx-expressing cells compared to that in the control groups, indicating that HBx facilitated the oxidative stress-related damage. To date, the mechanism of HBx-mediated liver cell death has primarily focused on non-inflammatory cell death, such as autophagy and apoptosis [23, 35]. Our current study shows, for the first time, a new form of cell death, HBx-induced pyroptosis, in normal hepatocytes exposed to H_2_O_2_. Pyroptosis refers to inflammatory cell death and may play a more significant role in HBV infection than other types of cell death. It is generally accepted that HBx can induce the production of pro-inflammatory cytokines in various liver cells. However, the underlying mechanistic details associated with HBx-induced inflammation remain unclear. Ample evidence suggests that NLRP3 inflammation occurs in hepatocytes, sinusoidal endothelial cells, and non-substantial cells, including Kupffer cells and hepatic stellate cells [8, 9, 36]. In comparison with that in other tissues, the expression level of caspase-1 in the liver is higher, and Kupffer cells can produce a large amount of IL-1β by activating NLRP3 [37]. Based on these data, pyroptosis may be a pivotal mechanism involved in HBx-mediated inflammation, at least in HL7702 liver cells.

Contrary to the findings of Yu et al., which state that HBV suppresses lipopolysaccharide (LPS)-mediated NLRP3 activation [38], we found LPS-induced NLRP3 activation, primarily in Kupffer cells. One reason for this discrepancy may be differences in study aims and design. For instance, they primarily focused on the hepatitis B e antigen (HBeAg), which inhibits NLRP3 activation. Moreover, although they conducted experiments using clinical samples, the sample size was relatively small, and they did not consider the effect of HBV DNA titer on NLRP3. We demonstrated that the inflammasome expression signal was enhanced in the liver tissue of patients with increased HBV DNA copy number.

Recently, some studies have showed that ASC can be released into the extracellular space from cells via inflammasome activation and accumulate in tissue. Then, ASC is phagocytosed by the surrounding immune cells, which promotes the production of pro-inflammatory cytokines and spread of inflammation [39, 40]. In addition, Franklin et al. demonstrated that subcutaneous and intraperitoneal injections of ASC cause acute inflammation in mice [41]. As ASC is a newly identified inflammatory protein that can mediate the spread of inflammation, we tested the serum levels of ASC in patients with various HBV DNA loads. Interestingly, higher serum ASC levels were observed in HBV-positive patients, similar to that observed in HBx-expressing cells. Collectively, these results suggest that HBx mediates the spread of inflammation via the promotion of ASC secretion. Further studies are needed to explore the mechanism of inflammation mediated by ASC.

Another study demonstrated that the NLRP3 inflammasome expression signal in normal, hepatitis-related, and cirrhotic tissue shows a continuously increasing trend; however, it becomes significantly reduced in hepatoma tissues [42]. Similarly, our data showed that inflammasome expression levels in liver cancer tissues from HBV-infected patients were significantly down-regulated compared with that in matched healthy para-cancer tissues (data not shown). Moreover, the mRNA and protein expression levels of the inflammasome in HepG2.2.15 cells, a human hepatoma cell line that stably expresses HBV, were decreased compared with those of control HepG2 cells (data not shown). The discrepant roles of HBV on the NLRP3 inflammasome may be dependent on the different stages of a natural HBV infection. It is possible that during the early stage of HBV infection, NLRP3 may be activated, resulting in inflammation and cell pyroptosis. Alternatively, long-lasting chronic inflammation may cause the development of liver cancer. When hepatocytes become cancerous, HBV may reduce the expression of the inflammasome and the secretion of pro-inflammatory mediators, thereby evading recognition and elimination by immune cells. Numerous studies have shown that the NLRP3 inflammasome can resist the formation of colon cancer [43], further supporting this speculation. However, the specific mechanism requires further investigation. Additionally, many studies showed that HMGB1 is an upstream activator of the inflammasomes [44, 45]. However, some studies have suggested that HMGB1 is a downstream protein of inflammasomes. Lamkanfi et al. showed that mice with knockout caspase 1 down-regulates LPS-induced HMGB1 production compared to wild-type mice. Also, the LPS-induced release of HMGB1 from macrophages depends on the activation of the NLRP3 inflammasome [46]. Hou et al. reported that pyroptosis enhances the secretion of HMGB1 in macrophages [47]. Chen et al. demonstrated that an adipokine (visfatin) can activate the NLRP3 inflammasome in vascular endothelial cells and promote the production of HMGB1, which leads to damage to the intercellular connections in the vascular endothelium [48]. Similarly, we observed that HBx increases the secretion of HMGB1 in hepatocytes under H_2_O_2_-stimulation, whereas NAC reduces the secretion of HMGB1 via inhibition of the activation of the NLRP3 inflammasome. The inconsistencies in the above results may be related to the complexity of cell signaling pathway regulation and the different types of cells and tissues.

While important new findings are provided, limitations in the current work have been noted. For instance, only a single human normal hepatic cell line was used in this study. Evaluation of other normal hepatic cell lines is, therefore, required. Also, the current study did not include any animal studies, which may provide relevant and essential insights.

In summary, under conditions of oxidative stress, HBx activated NLRP3 in normal hepatocytes and promoted pyroptosis via the mitochondrial ROS pathway, ultimately causing the release of ASC, IL-1β, IL-18, and HMGB1. Our study may provide novel insights into the mechanisms involved in HBV-induced hepatitis.

## Electronic supplementary material

Below is the link to the electronic supplementary material.

Supplementary material 1 (TIFF 31952 kb)

Supplementary material 2 (DOC 25 kb)
