# Supplementary figures and images for: Hepatitis B virus X protein promotes liver cell pyroptosis under oxidative stress through NLRP3 inflammasome activation
Source: Inflamm Res. 2020 Apr 28;69(7):683–96. doi: 10.1007/s00011-020-01351-z (PMC7261280; doi:10.1007/s00011-020-01351-z)

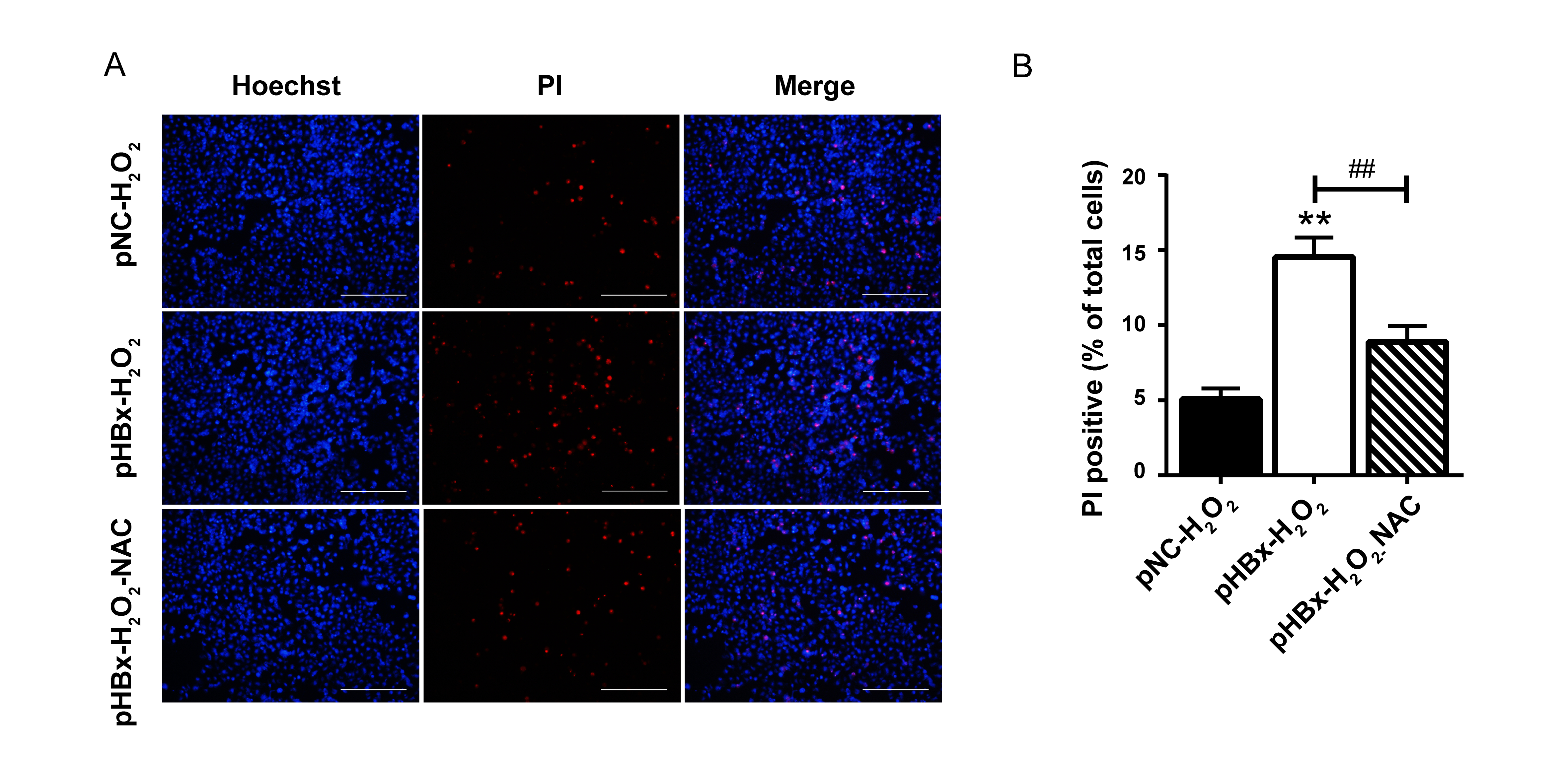

Supplement: Supplementary file 1 — Supplementary material 1 (TIFF 31952 kb) [file 11_2020_1351_MOESM1_ESM.tif]
