# Supplementary material for: Hepatitis B virus X protein promotes liver cell pyroptosis under oxidative stress through NLRP3 inflammasome activation
Source: Inflamm Res. 2020 Apr 28;69(7):683–96. doi: 10.1007/s00011-020-01351-z (PMC7261280; doi:10.1007/s00011-020-01351-z)
Supplement: Supplementary file 2 — Supplementary material 2 (DOC 25 kb) [file 11_2020_1351_MOESM2_ESM.doc]

Table 1 primers used for RT-PCR .

Gene Primer sequences

NLRP3 Forward 5’-GGTGGAGTGTCGGAGAAG-3’

Reverse 5’-CTGTCATTGTCCTGGTGTCT-3’

ASC Forward 5’-GCTGCTGGATGCTCTGTA-3’

Reverse 5’-AGGCTGGTGTGAAACTGAA-3’

Caspase-1 Forward 5’-GAGCAGCCAGATGGTAGAG-3’

Reverse 5’-CCCACAGACATTCATACAGTTTC-3’

IL-1β Forward 5’-TCACCTCTCCTACTCACT-3’

Reverse 5’-CGGTTGCTCATCAGAATG-3’

IL-18 Forward 5’-GACCTTCCAGATCGCTTCCTC-3’

Reverse 5’-GATGCAATTGTCTTCTACTGGTTC-3’

HMGB1 Forward 5’-TCAAAGGAGAACATCCTGGCCTGT-3’

Reverse 5’-CTGCTTGTCATCTGCAGCAGTGTT-3’

GSDMD Forward 5’-AGACCATCTCCAAGGAACTG-3’

Reverse 5’-GGACAACACCAGGCACTC-3’

GAPDH Forward 5’-GAAGGTGAAGGTCGGAGTC-3’

Reverse 5’-GAAGATGGTGATGGGATTTC-3’

NLRP3, NLR pyrin domain containing 3; ASC, apoptosis-associated speck-like protein containing a caspase recruitment domain (CARD); HMGB1, high mobility group box 1; GSDMD, gasdermin D; GAPDH, glyceraldehyde-3-phosphate dehydrogenase.
